# Cryo-EM structures of human SID-1 transmembrane family proteins and implications for their low-pH-dependent RNA transport activity

**DOI:** 10.1038/s41422-023-00893-1

**Published:** 2023-11-06

**Authors:** Le Zheng, Tingting Yang, Hangtian Guo, Chen Qi, Yuchi Lu, Haonan Xiao, Yan Gao, Yue Liu, Yixuan Yang, Mengru Zhou, Henry C. Nguyen, Yun Zhu, Fei Sun, Chen-Yu Zhang, Xiaoyun Ji

**Affiliations:** 1 National Key Laboratory of Pharmaceutical Biotechnology, School of Life Sciences, Chemistry and Biomedicine Innovation Center (ChemBIC), Institute of Artificial Intelligence Biomedicine, Nanjing University, Nanjing, Jiangsu China; 2 National Laboratory of Biomacromolecules, CAS Center for Excellence in Biomacromolecules, Institute of Biophysics, Chinese Academy of Sciences, Beijing, China; 3 University of Chinese Academy of Sciences, Beijing, China; 4 Shanghai Institute for Advanced Immunochemical Studies and School of Life Science and Technology, ShanghaiTech University, Shanghai, China; 5 Lingang Laboratory, Shanghai, China; 6 Shanghai Clinical Research and Trial Center, Shanghai, China; 7 Engineering Research Center of Protein and Peptide Medicine, Ministry of Education, Nanjing, Jiangsu China

**Keywords:** Cryoelectron microscopy, Molecular biology

Dear Editor,

In the nematode *Caenorhabditis elegans*, the systemic RNA interference defective protein 1 (SID-1) plays a crucial role in systemic RNA interference (RNAi) by facilitating the transport of exogenous double-stranded RNA (dsRNA) into the cytoplasm.^1^ Human SIDT1 and SIDT2 are closely related members of the SID-1 transmembrane family, and have been implicated in various biological processes such as glucose and lipid metabolism, innate immunity, and tumorigenesis.^2–6^ SIDT1 predominantly localizes to the plasma membrane and enhances the cellular uptake of synthetic small interfering RNA (siRNA)^7^ and plant-derived microRNA (miRNA).^8^ Using a *Sidt1*-knockout mouse model, our previous studies revealed that SIDT1 expressed by gastric pit cells facilitates the uptake of dietary miRNAs.^8^ Mechanistic analyses found that uptake of miRNAs by SIDT1 is lowpH-dependent.^8^ SIDT2 mainly localizes to the lysosomal membrane. It can facilitate the uptake of naked single-stranded (ss) oligonucleotides into cells,^9^ and mediate RNA/DNA uptake by lysosomes during RNautophagy/DNautophagy.^10,11^ However, molecular mechanisms underlying SIDT1- and SIDT2-dependent RNA uptake, especially for exogenous small RNAs, remain elusive. Here, we present the cryo-electron microscopy (cryo-EM) structures of human SIDT1 and SIDT2. Both structures reveal an overall architecture of a dimeric arrangement composed of an extracellular domain (ECD) rich in β-strands and a transmembrane domain (TMD) with 11 passes, highlighting the remarkable structural congruence. In situ assays confirmed that both proteins exist as dimers or higher-order oligomers. Importantly, the ECDs of SIDT1 and SIDT2 bind synthetic plant-derived miRNA only under acidic conditions. RNA binding under low pH can trigger higher-order assembly of the ECD dimer, suggesting the potential importance of oligomerization during RNA uptake. These findings provide insights into the lowpH-dependent activation of RNA-binding by SIDT1 and SIDT2, fostering a better understanding of nucleic acid delivery mechanisms.

We first solved cryo-EM structures of SIDT1 and SIDT2 at overall resolutions of 3.33 Å and 3.17 Å, respectively (Supplementary information, Figs. S1–S3 and Table S1). The electron density maps exhibited well-defined main-chain connectivity and side-chain densities in the ECDs, while the TMDs have relatively lower quality and resolution (Supplementary information, Fig. S4a–d). Both SIDT1 and SIDT2 form homodimeric structures involving their ECD and TMD, exhibiting a high degree of symmetry with a two-fold rotational axis perpendicular to the membrane surface. Blue native gel assays with full-length SIDT1 and SIDT2 also confirmed their dimeric conformation (Supplementary information, Fig. S1e). Bioinformatics analysis predicted eight *N*-glycosylation sites in SIDT1, with three glycosylation sites resolved in the EM map (Fig. 1a). The SIDT1 ECD consists of two subdomains, ECD1 and ECD2, connected by a flexible loop. A disulfide bond between C130 of ECD1 and C222 of ECD2, along with another pair of disulfide bonds between C212 and C271 on ECD2, contributes to the stability of SIDT1 (Supplementary information, Fig. S4e). SIDT2 also contains ten predicted *N*-glycosylation sites, with all six glycosylation sites on its ECD well resolved in the EM map, along with the corresponding disulfide bonds (Fig. 1a). The TMDs of both proteins consist of 11 transmembrane helices and feature two pairs of conserved disulfide bonds that are essential for structural integrity (Supplementary information, Fig. S4f). Indeed, the conservation of disulfide bonds extends to the entire SID-1 transmembrane family proteins (Supplementary information, Fig. S5). Notably, both SIDT1 and SIDT2 exhibit a potential zinc-binding site with three histidine residues within TMDs (Supplementary information, Fig. S4g, h), which is responsible for lipid hydrolytic activity.^12^ The ceramidase activity of both SIDT1 and SIDT2 was also confirmed in our biochemical assays (Supplementary information, Fig. S6). These structural similarities highlight the homology and functional relevance between SIDT1 and SIDT2.

**Fig. 1:**
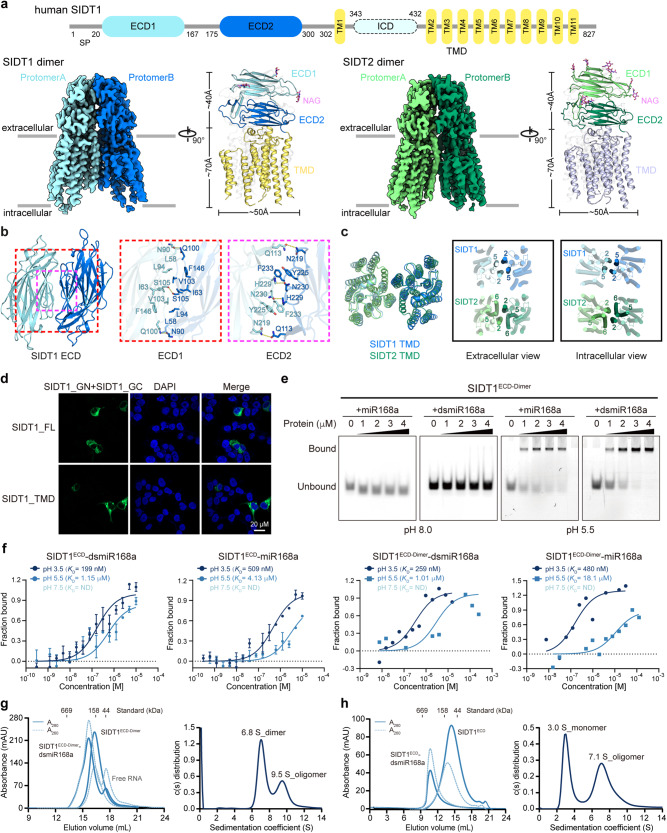
Structures of SIDT1 and SIDT2 homodimer and their pH-dependent RNA-binding and oligomerization. **a** Overall cryo-EM maps and structure models of SIDT1 and SIDT2. Protomers A and B of SIDT1 are colored in light blue and deep blue, respectively. They are colored in light green and deep green for SIDT2, respectively. **b** Detailed dimeric interactions of ECD1 and ECD2 between two SIDT1 protomers. Potential hydrophobic interacting residues are shown as sticks. Potential hydrogen bonds are represented as yellow dashed lines. **c** Comparison of dimer interface of the TMDs between SIDT1 and SIDT2. **d** High-resolution confocal images of the BiFC assay for SIDT1 (FL full-length, top; TMD, bottom) fused to the N-terminal and C-terminal fragments of GFP fluorescent protein. DAPI (blue) was used for nuclear staining. Scale bar, 20 µm. **e** EMSA results of SIDT1^ECD-Dimer^ under pH 5.5 and pH 8.0 conditions. Final protein concentrations in lanes 1–5 are 0, 1, 2, 3, and 4 μM, respectively, and final concentrations of the 5’-FAM-labeled small RNAs are 2.5 μM in each lane. Bound, protein-RNA complexes; Unbound, free RNA. **f** MST analysis measuring binding affinities of SIDT1^ECD^ and SIDT1^ECD-Dimer^ with small RNAs across different pH conditions. The calculated dissociation constant (*K*_D_) value represents affinity between SIDT1^ECD^ or SIDT1^ECD-Dimer^ with miRNAs. MST results are representative of three independent experiments. SEC (left panel) and SV-AUC (right panel) analyses of SIDT1^ECD-Dimer^ (**g**) and SIDT1^ECD^ (**h**) forming complexes with dsmiR168a, respectively. Experiments were performed at pH 5.5. Experiments were conducted twice. Data represent means ± s.d.

A comparative analysis of the interaction characteristics within the ECD and TMD regions of SIDT1 and SIDT2 was conducted. The dimer interface of the SIDT1 ECD can be divided into two regions (Fig. 1b). The first region involves hydrophobic interactions between ECD1 subdomains of two protomers, supported by hydrogen bond interactions (Fig. 1b). A conserved hydrogen bond between S105 residues is observed in SIDT1, corresponding to S92 in SIDT2. The second region involves a long loop on ECD2, connecting two β-strand sheets, with a potential hydrogen bond between H229 and N230 (Fig. 1b). This interaction closely resembles that observed in SIDT2, although the histidine is replaced by an asparagine (N214 in SIDT2). In addition, Q113 in SIDT1 may maintain a hydrogen bond interaction with N219 from the other protomer instead of the ionic bond interaction observed between R100 and D204 in SIDT2. Furthermore, SIDT2 features an additional β-strand (residues 27–33) involved in interactions at the dimer interface, while the corresponding region in SIDT1 remains unstructured, indicating a high level of flexibility and fewer interactions at the dimer interface of SIDT1 compared to SIDT2. In the SIDT2 ECD model, the dimerization interaction involves a buried surface area of ~1700 Å^2^ from each protomer, in contrast to ~1300 Å^2^ in the SIDT1 model, indicating differences in the proximity of the formed homodimers between SIDT1 and SIDT2, probably due to sequence variations.

Regarding the TMD, while the overall arrangement is similar in both SIDT1 and SIDT2, different transmembrane helices (TMs) contribute to dimerization (Fig. 1c). In SIDT1, the dimer interface primarily involves TM2 interacting with TM5 from the other protomer (Fig. 1c) and is driven by extensive hydrophobic interactions. TM6 does not appear to play a significant role in the SIDT1 dimer interface. In contrast, SIDT2 relies more on interactions between TM2 and TM6 for dimerization (Fig. 1c). Overall, while the ECD structure exhibits consistency, the individual helices of the TMD in SID-1 transmembrane family members show flexibility, highlighting the dynamic nature of these proteins in performing specific functions in membranes.

To investigate the in situ oligomeric states of SIDT1 and SIDT2, we performed bimolecular fluorescence complementation (BiFC) assays in living cells (Supplementary information, Fig. S7a). Co-expression of SIDT1 or SIDT2 tagged with complementary fragments of green fluorescent protein (GFP) resulted in robust green fluorescence, indicating that both proteins have the ability to form dimers or oligomers. The truncated TMD proteins also exhibited GFP fluorescence, suggesting that the TMD alone is sufficient for dimerization or oligomerization (Fig. 1d; Supplementary information, Fig. S7b).

To further investigate their oligomeric states in solution, with a particular focus on contribution of the ECD in higher-order assembly, we performed a series of in vitro assays. Truncated ECD domains of SIDT1 (SIDT1^ECD^) and SIDT2 (SIDT2^ECD^) separated by size exclusion chromatography (SEC) have different elution volumes, indicating potentially different oligomerization status (Supplementary information, Fig. S7c, d). Sedimentation velocity analytical ultracentrifugation (SV-AUC) analysis further confirmed that SIDT1^ECD^ exists as a monomer, with a sedimentation coefficient of 2.8 S and a molecular weight of ~44.6 kDa, while SIDT2^ECD^ forms a dimer with a sedimentation coefficient of 4.8 S and a molecular weight of ~74.2 kDa (Supplementary information, Fig. S7c, d). The distinct ECD oligomerization states might be attributed to variations in amino acid composition and interactions at the dimer interface as shown in their structures. These combined in situ and in vitro results demonstrate that SIDT1 and SIDT2 can exist as dimers or oligomers, with the TMD playing a critical role in maintaining dimeric assemblies.

The ECDs of SID-1, SIDT1 and SIDT2 have been previously studied for their RNA-binding capacity, and it has been shown that SIDT1 and SIDT2 ECDs both bind long dsRNA, but do not bind dsRNA < 300-bp or < 100-bp, respectively.^13^ This conflicts with the findings that SID-1 transmembrane family proteins can facilitate the uptake of exogenous small RNAs. To address this discrepancy, electrophoretic mobility shift assay (EMSA) was performed using a synthetic 21-nt plant miRNA, miR168a, known to be internalized by SIDT1.^14^ We engineered a fusion protein of SIDT1^ECD^ with immunoglobulin fragment crystallizable region (Fc; SIDT1^ECD-Fc^) to enhance dimerization (referred to as SIDT1^ECD-Dimer^) (Supplementary information, Fig. S8a, b). EMSA results revealed that SIDT1^ECD-Dimer^ does not bind to single-stranded miR168a, miR168a duplex (dsmiR168a), or ss/dsDNAs at pH 8.0 but exhibited clear bindings at pH 5.5 (Fig. 1e; Supplementary information, Fig. S8c). SIDT1^ECD^ behaves similarly to SIDT1^ECD-Dimer^ (Supplementary information, Fig. S8d) and these results further demonstrated that both SIDT1^ECD-Dimer^ and SIDT1^ECD^ can bind various plant-derived miRNAs (Supplementary information, Fig. S8e, f). Similar binding properties were observed for SIDT2 (Supplementary information, Fig. S8g). Microscale thermophoresis (MST) analysis of SIDT1^ECD^ and SIDT1^ECD-Dimer^ showed a low-pH-dependent RNA-binding affinity for miR168a and dsmiR168a (Fig. 1f). At pH 3.5, SIDT1^ECD^ has dissociation constant (*K*_D_) values of 509 nM for miR168a and 199 nM for dsmiR168a, which increases to 4.13 μM and 1.15 μM at pH 5.5, respectively. At pH 7.5, the interactions are too weak and non-discernible. Similarly, SIDT1^ECD-Dimer^ shows high affinity binding at pH 3.5 (*K*_D_ values: 480 nM and 259 nM for miR168a and dsmiR168a, respectively), but reduced affinity at pH 5.5 (*K*_D_ values: 18.1 μM and 1.01 μM, respectively) (Fig. 1f). Protein stability measured by differential scanning fluorometry showed that both SIDT1 and SIDT2 ECDs are more stable under acidic conditions (Supplementary information, Fig. S8h), supporting their potential low-pH-dependent functions in the physiological acidic environment. Furthermore, analysis of surface electrostatic potential on the ECD structures revealed positively charged regions on the β-strand surfaces of SIDT1 and SIDT2 ECDs at pH 7.5, with increased charge density and expanded distribution at pH 5.5. At pH 3.5, nearly all ECD regions displayed a positive charge (Supplementary information, Fig. S9), supporting the observed low-pH-dependent RNA-binding abilities of SIDT1 and SIDT2. These findings suggest that the ECDs of SIDT1 and SIDT2 efficiently bind to small RNAs under acidic conditions.

To further investigate the molecular effects of the pH-dependent interactions between ECD and RNAs, analytical SEC and SV-AUC analyses were performed. Interestingly, acidic stimulation alone does not trigger higher-order oligomerization of SIDT1^ECD-Dimer^ and SIDT1^ECD^, as evidenced by consistent elution volumes and sedimentation coefficients at different pH values (Supplementary information, Fig. S10a–d). However, when SIDT1^ECD-Dimer^ was incubated with dsmiR168a at pH 5.5, a stable complex with an increased molecular weight was formed (Fig. 1g). An even more remarkable elution volume shift was observed when SIDT1^ECD^ was mixed with dsmiR168a (Fig. 1g), suggesting the potential formation of tetramers. SV-AUC analysis confirmed the presence of larger assemblies in these complexes (Fig. 1g). SIDT1^ECD^ bound to dsmiR168a resulted in a substantially increased sedimentation coefficient compared to the monomeric SIDT1^ECD^ (Fig. 1h). The cross-linked SIDT1^ECD^–dsmiR168a complex revealed the formation of oligomers in the presence of RNA (Supplementary information, Fig. S10e). Similar results were also obtained for SIDT2^ECD^ (Supplementary information, Fig. S10f). SEC experiments confirmed that cross-linking of distinct higher-order oligomers of SIDT1^ECD-Dimer^ and SIDT1^ECD^ had occurred in the presence of RNA (Supplementary information, Fig. S10g, h). These findings highlight the role of RNA in facilitating ECD oligomerization under acidic conditions, providing valuable insights into the mechanisms of RNA uptake by both SIDT1 and SIDT2.

Cryo-EM structures of SIDT1 and SIDT2 show highly conserved features despite only 57% sequence identity. Notably, clinically reported single nucleotide polymorphisms like V78M in SIDT1 potentially affect structural stability and nucleic acid uptake capability.^15^ Additionally, the F169T and P186L mutations in SIDT1, and F154T and P171L mutations in the SIDT2^13^ may also influence the structural stability of the ECD. In contrast, TMD displays increased flexibility, implying its dynamic arrangement on the cell membrane (Supplementary information, Fig S11). This inherent flexibility may contribute to the nucleic acid uptake capabilities of the SID-1 transmembrane family proteins. Previous studies suggested that both SIDT1 and SIDT2 ECDs bind long dsRNA but require higher protein concentrations than SID-1 ECD.^13^ However, our findings indicate that the acidic environment (pH < 5.5) enhances the binding abilities of both SIDT1 and SIDT2 ECDs to small RNAs, which is consistent with our previous RNA uptake experiments and provides a biochemical connection from binding to transmembrane transport of RNA.^8^ These results may explain how the acidic environment enhances RNA transport efficiency via SIDT1, providing a potential molecular basis for the uptake of small RNAs mediated by SIDT1 and SIDT2.

The protonation state of protein/RNA interface residues can significantly affect their interactions, leading to a pH-dependent property. The presence of histidine residues, which have a pKa of ~6.0, likely contributes to the pH-dependent affinity between the ECD and RNA, particularly at pH 5.5. In addition, conserved charged residues like Arg, Lys, Asp, and Glu were found on the ECD surface (Supplementary information, Fig. S5). Given the impact of low pH on histidine protonation, we propose that the ECD–RNA interaction primarily occurs at the back-to-dimer interface. SIDT1^ECD^ binding to small RNAs under acidic conditions triggers higher-order oligomerization, which may play a crucial role in SIDT1-mediated RNA uptake and transport. This oligomerization tendency, while not observed in cryo-EM structures, might involve interactions with additional proteins or specific lipids (Supplementary information, Fig. S12). Furthermore, the ceramidase activity of both SIDT1 and SIDT2 may influence the fluidity of cell membrane system,^12^ which might also contribute to their role in facilitating transport of nucleic acids. In summary, our current study provides structural insights into the mechanistic understanding of roles of SIDT1 and SIDT2 in RNA uptake and intracellular trafficking.

### Supplementary information

Supplementary information

## Data Availability

The coordinates and EM map files for the SIDT1-overall, SIDT1-ECD, SIDT1-TMD, SIDT2-overall, SIDT2-ECD and SIDT2-TMD have been deposited in the Protein Data Bank (PDB) and the EM Data Bank (EMDB) under accession numbers PDB-8K13, PDB-8K1B, PDB-8K1D, PDB-8K10, PDB-8K11 and PDB-8K12, and EMDB-36785, EMDB-36791, EMDB-36792, EMDB-36782, EMDB-36783 and EMDB-36784, respectively.
