# Supplementary material for: Cryo-EM structures of human SID-1 transmembrane family proteins and implications for their low-pH-dependent RNA transport activity
Source: Cell Res. 2023 Nov 6;34(1):80–3. doi: 10.1038/s41422-023-00893-1 (PMC10770124; doi:10.1038/s41422-023-00893-1)
Supplement: Supplementary file 1 — Supplementary information [file 41422_2023_893_MOESM1_ESM.pdf]

Supplementary Information for

Cryo-EM structures of human SID-1 transmembrane family proteins and implications for their low-pH-dependent RNA transport activity

Le Zheng[1*], Tingting Yang[1*], Hangtian Guo[1*]†, Chen Qi[2,3*], Yuchi Lu[4,5*], Haonan Xiao[1], Yan Gao[4,6], Yue Liu[1], Yixuan Yang[1], Mengru Zhou[1], Henry C. Nguyen[1], Yun Zhu[2]†, Fei Sun[2,3]†, Chen-yu Zhang[1]† & Xiaoyun Ji[1,7]†

[1]The State Key Laboratory of Pharmaceutical Biotechnology, School of Life Sciences, Chemistry and Biomedicine Innovation Center (ChemBIC), Institute of Artificial Intelligence Biomedicine, Nanjing University, Nanjing, China.

[2]National Key Laboratory of Biomacromolecules, CAS Center for Excellence in Biomacromolecules, Institute of Biophysics, Chinese Academy of Sciences, Beijing 100101, People's Republic of China.

[3]University of Chinese Academy of Sciences, Beijing, China.

[4]Shanghai Institute for Advanced Immunochemical Studies and School of Life Science and Technology, ShanghaiTech University, Shanghai 201210, China.

[5]Lingang Laboratory, Shanghai, 200031, China

[6]Shanghai Clinical Research and Trial Center, Shanghai 201210, China.

[7]Engineering Research Center of Protein and Peptide Medicine, Ministry of Education, China.

† Correspondence: Hangtian Guo (guohangtian@nju.edu.cn) or Yun Zhu (zhuyun@ibp.ac.cn) or Fei Sun (feisun@ibp.ac.cn) or Chen-yu Zhang (cyzhang@nju.edu.cn) or Xiaoyun Ji (xiaoyun.ji@nju.edu.cn).

*These authors contributed equally: Le Zheng, Tingting Yang, Hangtian Guo, Chen Qi, Yuchi Lu.

Materials and methods

Recombinant construct preparation

The full-length cDNAs for human SIDT1 and SIDT2 (Uniport: Q9NXL6 and Q8NBJ9, respectively) were codon-optimized and synthesized by GenScript Co., Ltd. To facilitate protein expression and structural analysis, we constructed internal truncated constructs of SIDT1 (SIDT1$^{\Delta ICD}$, residues 366-408 deletion) and SIDT2 (SIDT2$^{\Delta ICD}$, residues 366-410 deletion) in the pFastBac1 vector containing an N-terminal efficient signal peptide derived from the influenza virus hemagglutinin HA (MKANLLVLLCALAAADA) and a triple Flag tag sequence downstream of the coding region. Mutations were introduced using a two-step PCR-based strategy, followed by meticulous sequencing to confirm clone identities.

For the expression and purification of the ECD proteins of SIDT1 and SIDT2, we subcloned the DNA sequences corresponding to amino acids 23-310 of SIDT1 and 22-292 of SIDT2 into a customized pMlink vector. This vector contains an N-terminal melittin signal sequence (MKFLVNVALVFMVVYISYIYA) and a C-terminal 6 × His-tag. To ensure stability, we utilized a fusion strategy by adding an immunoglobulin fragment crystallizable region (Fc fragment) to the C-terminus of the ECD to facilitate dimerization. We also introduced a 6 × His-tag and a short GS linker. The final construct was subcloned into the pMlink expression vector, and clone identities were verified by Sanger sequencing.

Expression and purification of full-length SIDT1 and SIDT2

The full-length SIDT1 and SIDT2 were expressed using the Bac-to-Bac Baculovirus Expression System (Invitrogen). The *Sf9* cells were cultured in Sf-900 II SFM (Gibco, USA). Bacmid DNAs were generated and transfected into *Sf9* cells to produce and amplify baculovirus. A total of 6-8 liters of infected *Sf9* cells were harvested 60 hours post-infection and then lysed in lysis buffer containing 50 mM HEPES pH 7.5, 300 mM NaCl, and supplemented with cocktail inhibitor (1.04 mM AEBSF, 0.8 μM Aprotinin, 50 μM Bestatin, 15 μM E-64, 20 μM Leupeptin and 15 μM Pepstatin A, MCE, Cat#

HY-K0010) and 1 mM PMSF. Cells were disrupted using high-pressure cell disruption at 800 bars. The cell debris was removed by low-speed centrifugation for 30 minutes and the supernatant was further subjected to high-speed centrifugation at 150,000 × g for 120 minutes using a Ti-45 rotor (Beckman). Then the membrane component was resuspended and homogenized using a glass dounce homogenizer for 20 cycles on ice in solubilization buffer containing 50 mM HEPES pH 7.5, 300 mM NaCl, 2% (*w/v*) *n*-dodecyl-β-D-maltoside (DDM, Anatrace), 0.2% (*w/v*) cholesteryl hemisuccinate (CHS, Anatrace), cocktail inhibitor and solubilized at 4 °C with gentle agitation for 2 hours. The extraction was centrifuged at 39,000 × g (Beckman) for 60 minutes to remove insoluble components, and the supernatant was incubated with anti-Flag affinity resin (GenScript Co., Ltd.) at 4 °C for 90 minutes. The resin was pooled and rinsed with 50 mL of buffer A containing 50 mM HEPES pH 7.5, 300 mM NaCl, 0.02% (*w/v*) lauryl-maltose-neopentyl-clycol (LMNG, Anatrace) and 0.002% (*w/v*) CHS; 30 mL of buffer B containing 50 mM HEPES pH 7.5, 300 mM NaCl, 2 mM ATP, 10 mM $MgCl_2$,0.02% (*w/v*) LMNG and 0.002% (*w/v*) CHS (Anatrace); 30 mL of buffer C containing 50 mM HEPES pH 7.5, 300 mM NaCl,0.01% (*w/v*) LMNG and 0.001% (*w/v*) CHS. The protein was eluted using a buffer containing 50 mM HEPES pH 7.5, 300 mM NaCl, 0.01% LMNG, 0.001% CHS, and 500 μg/ml synthesized FLAG peptide (GenScript Co., Ltd.). The eluted protein was further purified by SEC using a Superose 6 increase 10/300 column (GE Healthcare, USA) pre-equilibrated with running buffer containing 25 mM HEPES pH 7.5, 150 mM NaCl, 0.5 mM EDTA, 0.01% (*w/v*) LMNG and 0.001% (*w/v*) CHS. The purity of the proteins was confirmed by SDS-PAGE and Coomassie blue staining. Peak fractions containing the target proteins were pooled, concentrated to a final concentration of 3.5 mg/mL using Millipore 50-kDa cut-off filters (Millipore, USA), and subsequently flash-frozen in liquid nitrogen for cryo-EM analysis.

Expression and purification of secreted SIDT1 and SIDT2 extracellular domain

The $SIDT1^{ECD}$ and $SIDT2^{ECD}$ proteins were expressed using FreeStyle 293-F cells (Invitrogen). The cells were cultured in OPM-293 CD05 medium (OPM Biosciences Co., Ltd.) at 37 °C with 5% CO_2 in a ZCZY-CS8 shaker (Shanghai Zhichu Instrument

Co., Ltd.) at 120 rpm. Transfection was performed when the cell density reached 2.5 × 10^6 cells per mL by using expression plasmids and polyethylenimines (PEIs, (Polysciences, USA). Approximately 1.5 mg of plasmids were premixed with 4.5 mg of PEIs in 50 mL of fresh medium for 25 minutes before application. Transfected cells were harvested 72 hours post-transfection and the 6 × His-tagged protein in the supernatant was purified using Ni-NTA (GE Healthcare, USA) affinity chromatography. The resin was washed with buffer A containing 50 mM HEPES pH 7.5, 300 mM NaCl, 20 mM imidazole, and the target protein was eluted with buffer containing 50 mM HEPES pH 7.5, 300 mM NaCl and 250 mM imidazole. The purified protein was concentrated and subjected to gel-filtration chromatography using a Superdex 200 increase 10/300 column (GE Healthcare, USA), pre-equilibrated with gel-filtration buffer containing 25 mM HEPES pH 7.5, 200 mM NaCl.

Single-particle Cryo-EM sample preparation and data collection

The purified SIDT1 sample (3 μL) was applied onto an H_2/O_2 glow-discharged, 300-mesh R0.6/1.0 holey carbon copper grid (preprocessed with 0.1% poly L-lysine hydrobromide after glow-discharging) (Quantifoil), or an R1.2/1.3 amorphous nickel-titanium alloy (ANTA) film grid (Nanodim)[1], respectively. The grid was blotted for 2.5 s with a blot force of -1 for the copper grid or 3 s with a blot force of 0 for the ANTA grid, both at 8 °C and 100% humidity. Finally, the grids were plunge-frozen in liquid ethane using a Vitrobot Mark IV (ThermoFisher Scientific). Cryo-EM datasets were collected using a 300 kV Titan Krios microscope (ThermoFisher Scientific) equipped with a K3 Summit detector (Gatan), or a K2 Summit detector with a GIF Quantum energy filter. The micrographs were automatically collected using SerialEM[2] in super-resolution mode, with a nominal magnification of 29 k × (105 k × for the ANTA dataset). For the copper grid dataset, the exposure time was set to 2.4 s with a total accumulated dose of 60 electrons per $Å^2$, resulting in a final pixel size of 0.82 Å, and a total of 9,115 micrographs were collected with a defocus range comprised between -1.2 and -2.0 μm. For the ANTA dataset, the exposure time was set to 11.02 s with a total accumulated dose of 60 electrons per $Å^2$, which yields a final pixel size of 0.82 Å, and a total of

4,445 micrographs were collected with a defocus range comprised between -1.2 and -1.8 μm.

The data collection strategy for SIDT2 was consistent with that for SIDT1, resulting in 4,917 micrographs for the copper dataset and 5,547 (2,387 + 3,160) micrographs for the ANTA photographs, respectively.

The statistics of cryo-EM data collection are summarized in Supplementary information, Table S1.

Cryo-EM data processing

All dose-fractioned images were motion-corrected and dose-weighted by MotionCorr2 software[3] and their contrast transfer functions (CTF) were estimated using Gctf[4] in RELION[5]. The following particle picking, extraction, 3D classification and 3D refinements were carried out in RELION, while the initial 2D classification, the final non-uniform refinement and the local resolution estimation were performed using cryoSPARC[6]. For SIDT1, a total of ~3,854 k particles were extracted for the subsequent 2D classification. After several rounds of 3D classification, a ~4.1 Å map with clear TMDs and ECDs was generated. The remaining 249,851 particles were further classified, with 149,089 particles being re-extracted with a pixel size of 1.35 Å. These particles were polished with C2 symmetry, generating a density map with an overall resolution of 3.75 Å, which has been further refined to 3.33 Å using the non-uniform refinement and local refinement in cryoSPARC. Based on this, we create masks to realign and optimize the ECD and TMD regions, respectively. After non-uniform refinement and local refinement with C2 symmetry, we obtained a local optimization map for ECD with an overall resolution of 3.47 Å and a local optimization map for TMD with an overall resolution of 3.60 Å.

The data processing strategy for SIDT2 was consistent with that for SIDT1. A total of ~3,164 k (~1,694 k from the copper grid dataset and ~1,470 k from the ANTA grid dataset) particles were extracted from a total of 10,464 micrographs for subsequent classifications. Similarly, we obtained a final map using 201,729 particles with an

overall resolution of 3.17 Å, as well as two local-refined maps with an overall resolution of 3.27 Å for ECD and 3.33 Å for TMD, respectively.

The full cryo-EM data processing workflow is described in Supplementary information, Fig. S2 and Fig. S3.

Model building and structure refinement

To build the SIDT1 dimer structure, an initial structure model for human SIDT1 predicted by AlphaFold[7] was placed and rigid-body fitted into the cryo-EM electron density maps using UCSF Chimera[8]. The ECD and TMD regions of SIDT1 were individually built based on two local-refined maps wherein these regions were better resolved. Bulky residues such as Phe, Trp, Arg, Lys and Tyr were used as references for sequence assignment. The manual and automated model building were iteratively performed using Coot 0.9.6[9] and real-space refinement in Phenix 1.20[10].

The model building and structure refinement strategies for SIDT2 were consistent with that for SIDT1.

The data validation and model refinement statistics are summarized in Supplementary information, Table S1.

Bimolecular fluorescence complementation

The GFP-based BiFC technique was used to investigate the SIDT1 and SIDT2 oligomerization in living cells. Fusion constructs were generated using fragments derived from GFP, including GFP fragment 1 (GN, amino acids 1-173) and fragment 2 (GC, amino acids 155-238)[11]. Full-length SIDT1 and SIDT2 cDNAs were inserted upstream of the GN and GC fragments, respectively, to generate recombinant proteins (SIDT1/2_GN and SIDT1/2_GC). These fusion constructs were connected by a Flag tag and a 10-amino acid linker encoding $(GGGGS)_2$. The coding sequences of the recombinant proteins were then inserted into the *BamHI* and *EcoRI* sites of the pcDNA3.1(+) vector (Invitrogen), and all clones were verified through DNA sequencing.

Monolayers of HEK293T cells cultivated on coverslips were co-transfected with equimolar amounts of SIDT1/2_GN and SIDT1/2_GC plasmids using Lipofectamine 3000 (Invitrogen, USA). After 24 hours of transfection, the culture media was removed, and the coverslips were washed with PBS. Subsequently, the cells were fixed with 4% paraformaldehyde at room temperature (RT) for 10 minutes, followed by a 10-minute incubation with 0.5% saponin to permeabilize the cells. The coverslips were then washed three times with PBS and stained with 2-(4-Amidinophenyl)-6-indolecarbamidine dihydrochloride (DAPI) for 10 minutes at RT. Images were acquired using a Zeiss LSM 980 scanning confocal microscope and processed with ZEN software (Carl Zeiss).

Electrophoretic mobility shift assay

The interaction between $SIDT1^{ECD}$, $SIDT1^{ECD\text{-}Dimer}$, and $SIDT2^{ECD}$ with various miRNA fragments was investigated using EMSA. A forward ssRNA oligonucleotide labeled with Carboxyfluorescein (FAM) at the 5' end was synthesized by GenScript Co., Ltd. and annealed with an equimolar amount of the reverse strand. For the EMSA, increasing amounts of protein (1, 2, 3, 4, and 5 μM) were incubated with approximately 2.5 μM FAM-labeled miRNA or dsmiRNA at pH 5.5. EMSA reaction buffer containing 30 mM NaCl, 40 mM Tris (pH 5.5, adjusted with acetate), 2.5 mM EDTA, and 5% glycerol (*v/v*) or 30 mM NaCl, 25 mM Tris-HCl (pH 8.0), 192 mM Glycine, 5% glycerol (*v/v*) for a total volume of 20 μL at RT for 30 minutes. The resulting products were then separated on 5% native acrylamide gels (37.5:1 for acrylamide: bisacrylamide) in 1 × Tris-acetate-EDTA (TAE) running buffer (pH 5.5) or 1 × Tris-Glycine (TG) running buffer (pH 8.0)[12] under an electric field of 10 V/cm for about 1 hour on ice. The gel was visualized and analyzed using the Tanon-5100 Fluorescent Imaging System (Tanon Science & Technology).

Size exclusion chromatography

All proteins employed in biochemical assays were purified as previously described. We utilized SEC to assess $SIDT1^{ECD}$ and $SIDT2^{ECD}$ oligomerization. The SEC analyses

were performed with a Superose 6 increase 10/300 column (GE Healthcare, USA) or a Superdex 200 increase 10/300 column (GE Healthcare, USA) in the buffer containing 25 mM MES pH 5.5, 100 mM NaCl. The peak fractions of targets were confirmed by SDS-PAGE followed by Coomassie blue staining. Data analysis was performed using GraphPad Prism 9 (GraphPad Software, San Diego, CA, USA).

Sedimentation velocity analytical ultracentrifugation

We used SV-AUC to determine the sedimentation coefficients of $SIDT1^{ECD}$, $SIDT2^{ECD}$, $SIDT1^{ECD\text{-}Dimer}$, $SIDT1^{ECD}$-dsRNA, and $SIDT1^{ECD\text{-}Dimer}$-dsRNA, and to calculate their molecular weights. The SV-AUC experiments were performed in a Beckman Coulter XL-I analytical ultracentrifuge (Beckman Coulter Inc., USA) using a two-channel centerpiece equipped with an An-50 Ti rotor (Beckman Coulter Inc., USA). Each sample was diluted to achieve a final volume of 400 µL (with an A280 nm absorption of approximately 1.0) in buffers with varying pH levels: a pH 3.5 buffer containing 25 mM sodium acetate and 100 mM NaCl, a pH 5.5 buffer containing 25 mM MES and 100 mM NaCl, and a pH 7.5 buffer containing 25 mM HEPES and 100 mM NaCl. Samples were loaded into 120-mm double-sector aluminum centerpieces and run at a rotor speed of 40,000 rpm under vacuum. Absorbance data were collected at 20 °C, with simultaneous measurements at 280 nm and 260 nm for the apo-form and oligonucleotide-bound complex, respectively. SV-AUC data were globally analyzed using the SEDFIT program[13] and fitted to a continuous c(s) distribution model to determine the sedimentation coefficients and molecular mass of each peak. All data were generated using GraphPad Prism 9 and Illustrator software, and c(s) and molecular weight were plotted.

Microscale Thermophoresis

The MST experiments were conducted using a Monolith NT.115 instrument (NanoTemper Technologies). Purified $SIDT1^{ECD}$ and $SIDT1^{ECD\text{-}Dimer}$ were first exchanged into a labeling buffer containing 25 mM $NaHCO_3$ pH 8.3, 100 mM NaCl, and 0.05% Tween-20. After buffer exchange, the proteins were labeled with a RED-

NHS Labeling Kit (NanoTemper Technologies) according to the manufacturer's instructions. For the MST measurements, the labeled proteins were dialyzed and exposed to various pH conditions using buffers: pH 3.5 (25 mM sodium acetate, 100 mM NaCl, and 0.05% Tween-20), pH 5.5 (25 mM MES, 100 mM NaCl, and 0.05% Tween-20), and pH 7.5 (25 mM HEPES, 100 mM NaCl, and 0.05% Tween-20). Throughout the experiments, the labeled protein concentration was maintained at 5 nM. 16-step serial dilutions of dsmiR168a or ssmiR168a were prepared in the same buffer to ensure consistent buffer conditions. MST measurements were performed at a constant temperature of 25 °C, with a 5-second LED on-time followed by a 30-second MST on-time. The LED and MST power settings were optimized for each experiment to achieve optimal signal-to-noise ratios and minimize aggregation or adsorption effects. Data were collected and analyzed using the MO. Affinity Analysis software (NanoTemper Technologies). To ensure the accuracy and reliability of the results, MST experiments were performed in triplicate, and the data were averaged to determine the final K_D values.

DSF

The thermal stability analysis was conducted using the Tycho NT.6 (NanoTemper Technologies). SIDT1^{ECD} and SIDT2^{ECD} were diluted to a final concentration of 0.25 mg/mL at different pH values (pH 7.5, 5.5, and 3.5) and analyzed in triplicates using capillary tubes. Protein unfolding was monitored by measuring the intrinsic fluorescence at wavelengths of 350 nm and 330 nm while gradually increasing the temperature from 35 to 95 °C at a rate of 30 K/minute. The obtained data was analyzed, smoothed, and the derivatives were calculated using the internal evaluation features of the Tycho instrument.

Cross-Linking Assay

SIDT1^{ECD} was placed in an acidic buffer with 20 mM MES pH 7.5 and 100 mM NaCl. Next, equimolar amounts of dsmiRNA168a and SIDT1^{ECD} were co-incubated at RT for 30 minutes. Following the incubation, glutaraldehyde crosslinking was carried out on

proteins, with and without dsmiRNA168a. $SIDT1^{ECD}$ was cross-linked in 20 mM MES pH 7.5, 100 mM NaCl, glutaraldehyde (GA) cross-linker (Macklin, Cat#C10366284) was resuspended to 10% (*v/v*) and the usage concentrations of glutaraldehyde (GA) include a gradient series: 0%, 0.05%, 0.1%, 0.2%, 0.5%. Cross-linking was allowed to proceed for 30 minutes at RT. Cross-linked samples were quenched in 0.1 M Tris-HCl pH 8.0, resolved by Western blot.

SIDT1 and SIDT2 ceramidase activity assay

SIDT1 and SIDT2 enzymatic activity were evaluated through Liquid Chromatography-Mass Spectrometry/Mass Spectrometry (LC-MS/MS) analyses for sphingosine detection and quantification, using the d-erythrosphingosine (d18:1) (Cat# 860490P, Avanti Polar Lipids) as the standard. Ceramidase activity was assessed by incubating 1.8 μM of purified SIDT1 and SIDT2 proteins with 40 μM ceramide (d18:1/18:0) (Cat# 860518P, Avanti Polar Lipids) at room temperature for 30 minutes, in a solution of 25 mM HEPES pH 7.5, 150 mM NaCl, 0.02% (*w/v*) DDM, and 0.002% (*w/v*) CHS. Methanol was added to quench reactions, achieving a final concentration of 30%. Ceramidase activity was then quantified by comparing peak areas with sphingosine standards. Lipids were extracted from reaction samples using the previously reported method[14]. The lipid extraction from samples proceeded by adding a dichloromethane/methanol/water (2.5:2.5:2 *v/v/v*) mixture and centrifuging. The collected organic phase was dried under nitrogen, redissolved in 30 μl of methanol, and stored at -20 °C until LC-MS analysis.

The LC-MS analysis was conducted using an AB SCIEX TripleTOF® 4600 System (SCIEX, Framingham, MA, USA). Sample separation was achieved on an absolute AQ-G8 column (particle size 3 μm, 2.1 × 100 mm) (Waters) maintained at a constant temperature of 40 °C. The mobile phases utilized were eluent A (containing 0.1% formic acid) and eluent B (comprised of acetonitrile). The gradient program was set as follows: 40% A and 60% B at 0 min, maintained at the same composition until 2 minutes, then changed to 70% B at 5 minutes, 95% B at 7 minutes, held at 95% B until 10 minutes, and returned to 40% A and 60% B at 10.1 minutes. The flow rate was

maintained at 0.4 mL/min. The auto-sampler temperature was set at 8 °C, and the injection volume was 5 μL. The mass spectrometer was operated in positive electrospray ionization mode. The instrumental parameters were set as follows: Ion Source Gas1 (GS1) and Ion Source Gas2 (GS2) were both set at 55 psi, Ion Spray Voltage Floating (ISVF) was set at 5500 V, the source temperature was 600 °C, the declustering potential was 80 V, and the collision energy was 10 eV. Data was captured in full scan mode over a mass range of 100-1300 m/z with a scan time of 0.18 s. For tandem mass spectrometry (MS/MS) analysis, the 8 most intense precursor ions from each survey scan were selected for subsequent fragmentation and analysis. Data processing was performed using Analyst® TF 1.7 Software (SCIEX, Framingham, MA, USA). Analytes were quantified by comparing the peak areas to those of an internal standard. System calibration and analysis of quality control samples were carried out regularly to ensure data reliability and maintain system stability throughout the analysis.

Quantification of the ceramidase activities of SIDT1 and SIDT2 was performed using d-erythrosphingosine (d18:1) as a standard through high-resolution LC-MS/MS analysis. LC-MS/MS analyses were performed using a Water ACQUITY HPLC system coupled with a QTRAP® 6500+ mass spectrometer (AB SCIEX, Framingham, MA, USA). Samples were injected onto a Water HSS T3 (2.1 × 100 mm, 1.8 μm) using an 8.5-minute linear gradient at a flow rate of 0.4 mL/min for the positive/negative polarity mode. The eluents were eluent A (0.1% Formic acid–water) and eluent B (0.1% Formic acid-acetonitrile). The solvent gradient was set as follows: 50% B, 1 minute; 50-95% B, 1-3 minutes; 95% B, 3-5 minutes; 95-50% B, 5-5.5 minutes; 50% B, 5.5-8.5 minutes. QTRAP® 6500 + mass spectrometer was operated in positive polarity mode with a Curtain Gas of 40 psi, Collision Gas of Medium, IonSpray Voltage of 5500 V, Temperature of 450 °C, Ion Source Gas of 1:55, Ion Source Gas of 2:55. The acquired data was controlled and processed using Analyst® TF 1.7 software (SCIEX, Framingham, MA, USA) for instrument control and Multiquant 3.03 software (SCIEX, Framingham, MA, USA) for MRM data processing, respectively.

Supplementary information, Fig. S1

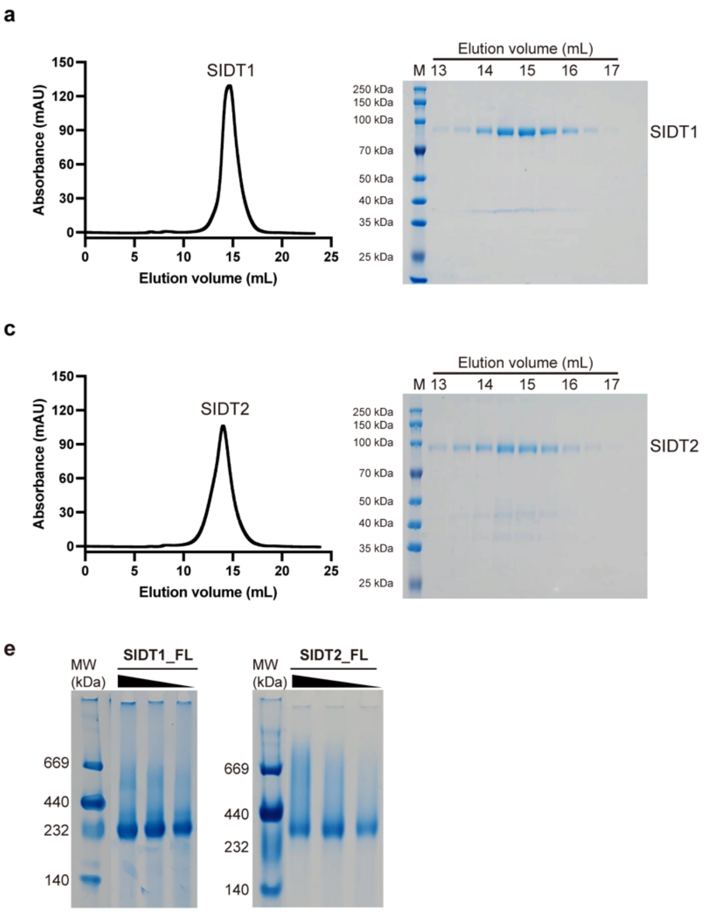

Supplementary information, Fig. S1 | Protein purification and cryo-EM analysis of human SIDT1 and SIDT2. a, c SEC profiles of SIDT1 (**a**) and SIDT2 (**c**) on a Superose 6 increase 10/300 column, respectively. Peak fractions were visualized by Coomassie blue staining. kDa, kilodaltons. M, marker. **b, d** Representative cryo-EM micrographs and 2D class averages of SIDT1 (**b**) and SIDT2 (**d**), respectively**.** The experiment was repeated independently more than three times with similar results. **c** BN-PAGE analysis of full-length SIDT1 and SIDT2, expressed in insect *Sf9* cells, reveals a homodimer formation in solution.

Supplementary information, Fig. S2

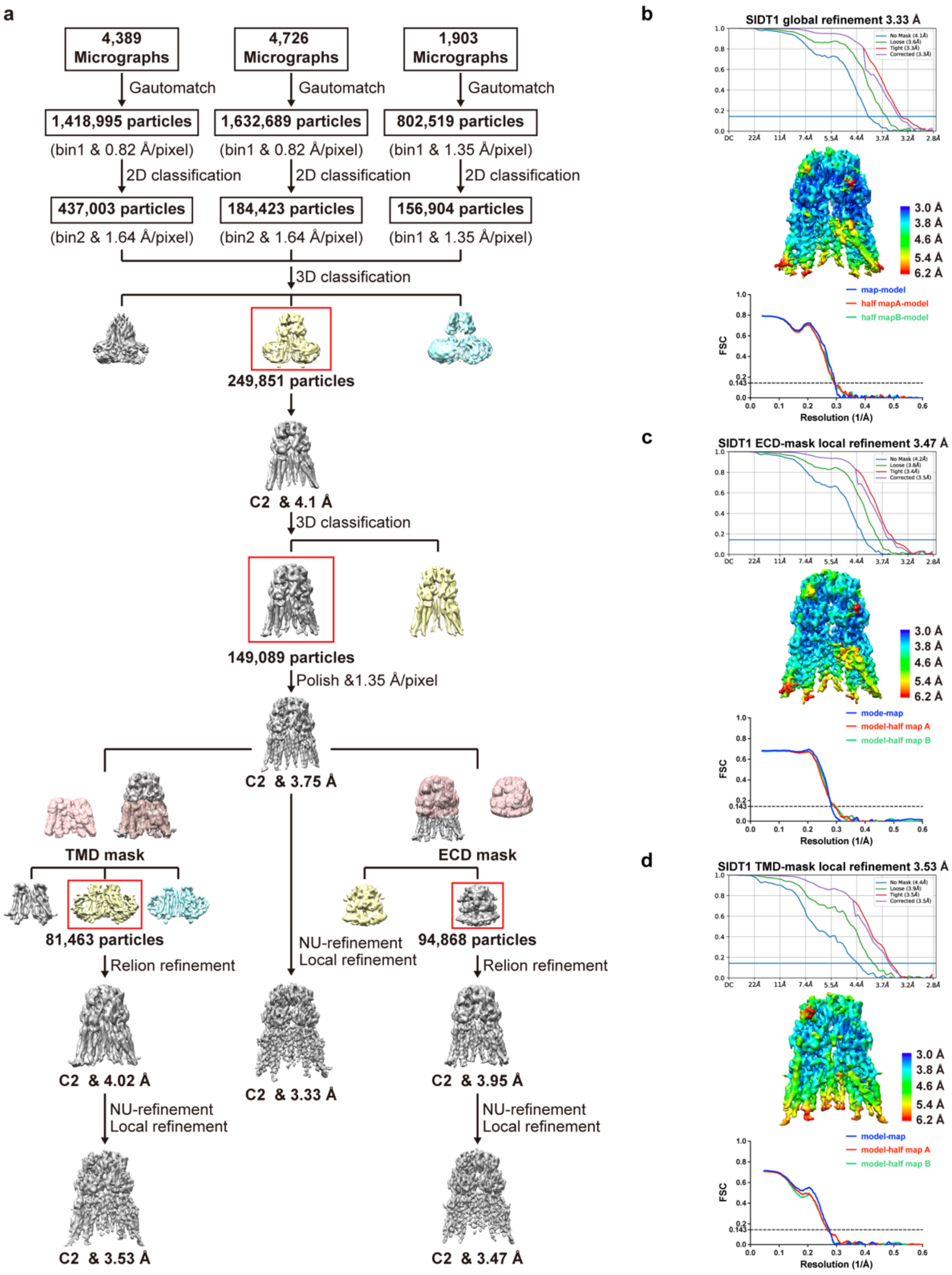

Supplementary information, Fig. S2 | Cryo-EM data processing and validation for SIDT1. a Workflow for the SIDT1 3D reconstructions. **b-d** The global FSC validation, cryo-EM density colored according to the local resolution and model-map FSC for the overall (**b**), local refinement with an ECD mask (**c**), and local refinement with a TMD mask (**d**) of SIDT1, respectively.

Supplementary information, Fig. S3

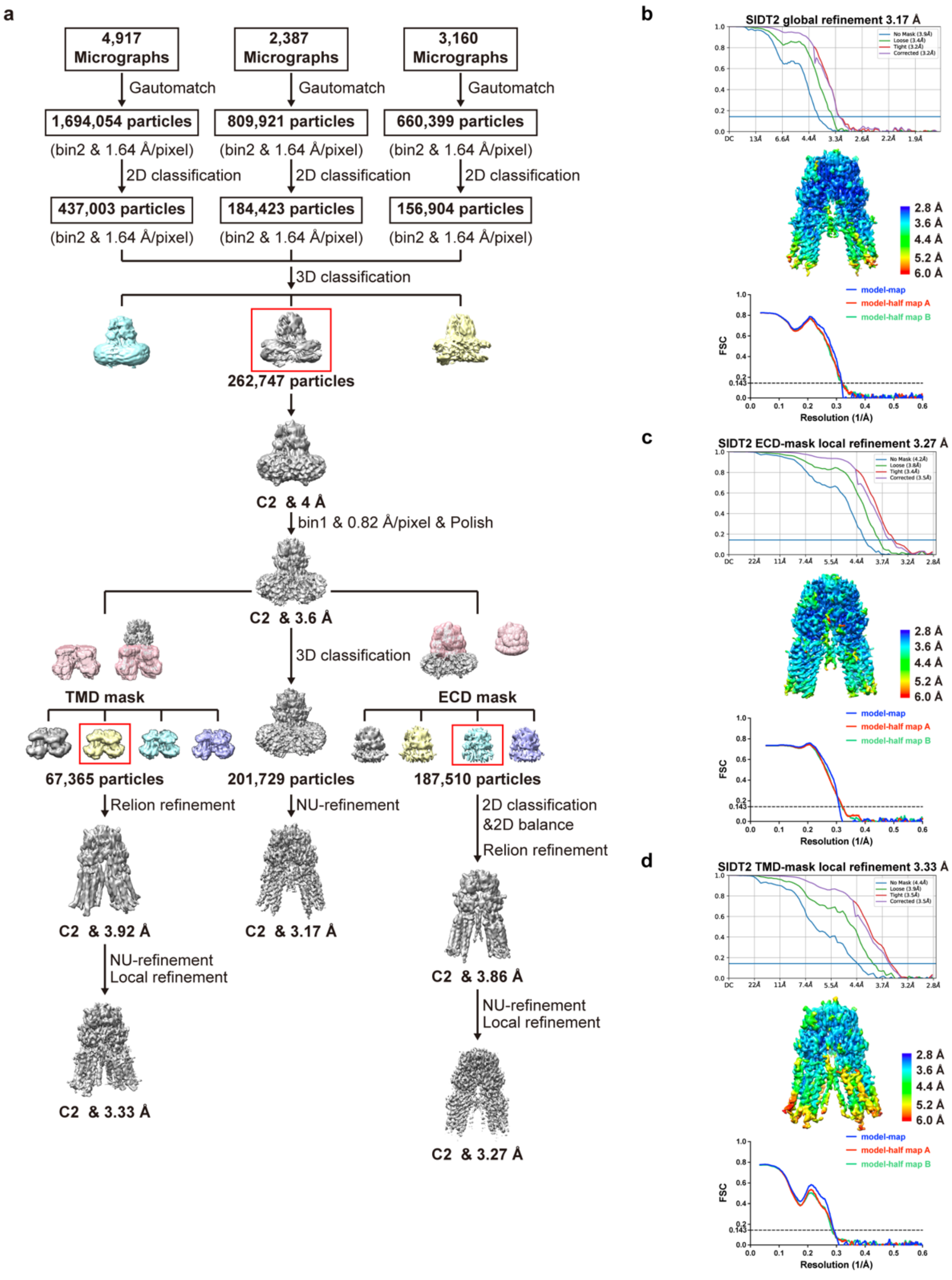

Supplementary information, Fig. S3 | Cryo-EM data processing and validation for SIDT2. a Workflow for the SIDT2 3D reconstructions. **b-d** The global FSC validation, cryo-EM density colored according to the local resolution and model-map FSC for the overall (**b**), local refinement with an ECD mask (**c**), and local refinement with a TMD mask (**d**) of SIDT2, respectively.

Supplementary information, Fig. S4

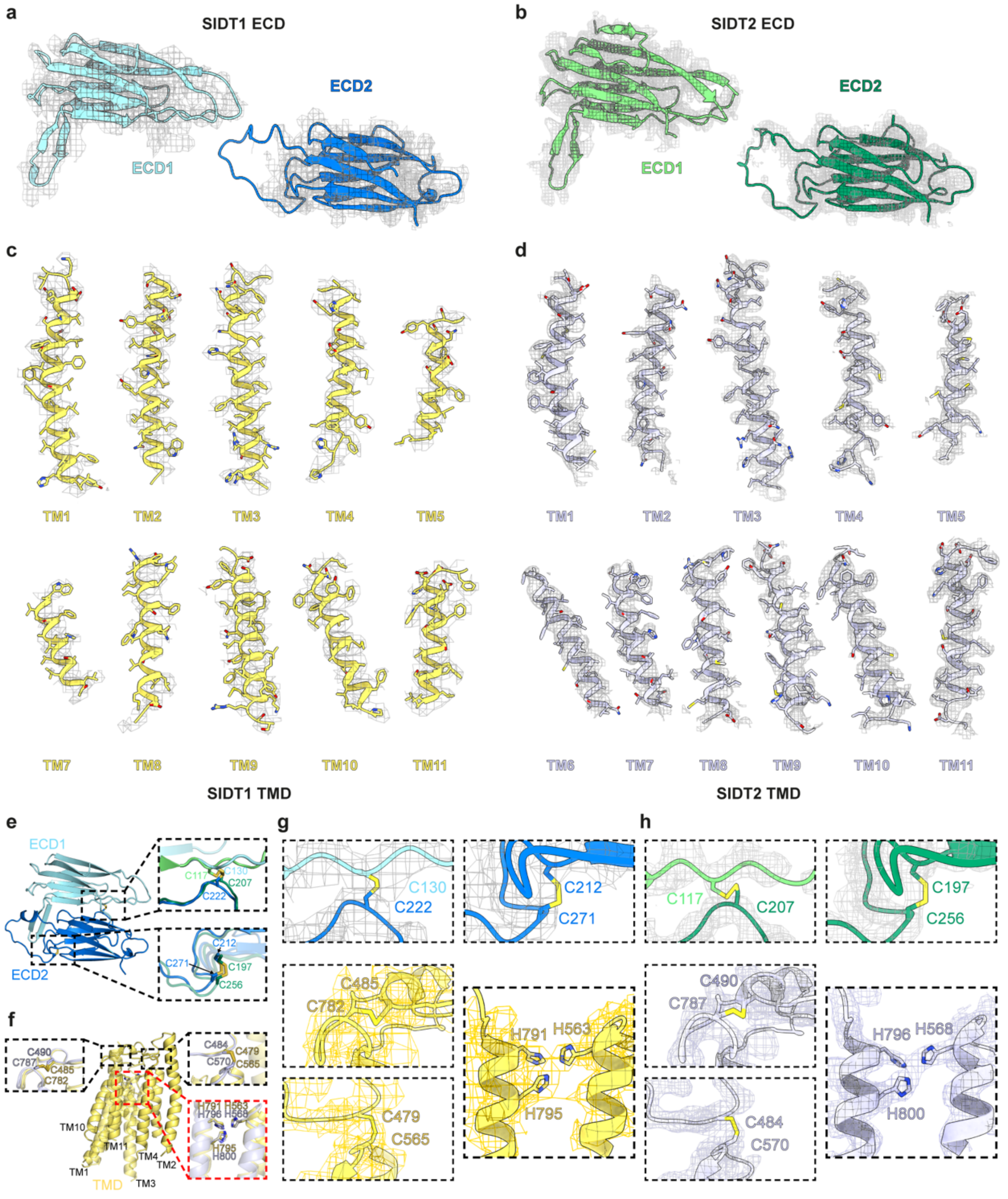

Supplementary information, Fig. S4 | Representative sharpened cryo-EM density maps of SIDT1 and SIDT2. a, b The EM densities of SIDT1 ECD (**a**) and SIDT2 ECD (**b**). **c, d** The EM densities of the TMD helices of SIDT1 (**c**) and SIDT2 (**d**). **e** A close-up view of the SIDT1 ECD. The SIDT1 ECD contains two subdomains, ECD1 and ECD2. Two pairs of conserved disulfide bonds that enhance the structural stability of the ECD are shown as sticks, along with the corresponding disulfide bonds in SIDT2. **f** A close-up view of the SIDT1 TMD. Two pairs of disulfide bonds that connected the

loop 2-3 to loop 4-5 and loop 10-11 are shown as sticks, along with the corresponding disulfide bonds in SIDT2. The putative Zn^{2+}-binding site is also conserved in SIDT1 and SIDT2 and the corresponding three histidine residues are shown as sticks. **g, h** The EM densities of two pairs of disulfide bonds in SIDT1 ECD (**g, upper panel**), SIDT2 ECD (**h, upper panel**), and two pairs of disulfide bond and the putative Zn^{2+}-binding site in SIDT1 TMD (**g, lower panel**)) and SIDT2 TMD (**h, lower panel**).

Supplementary information, Fig. S5

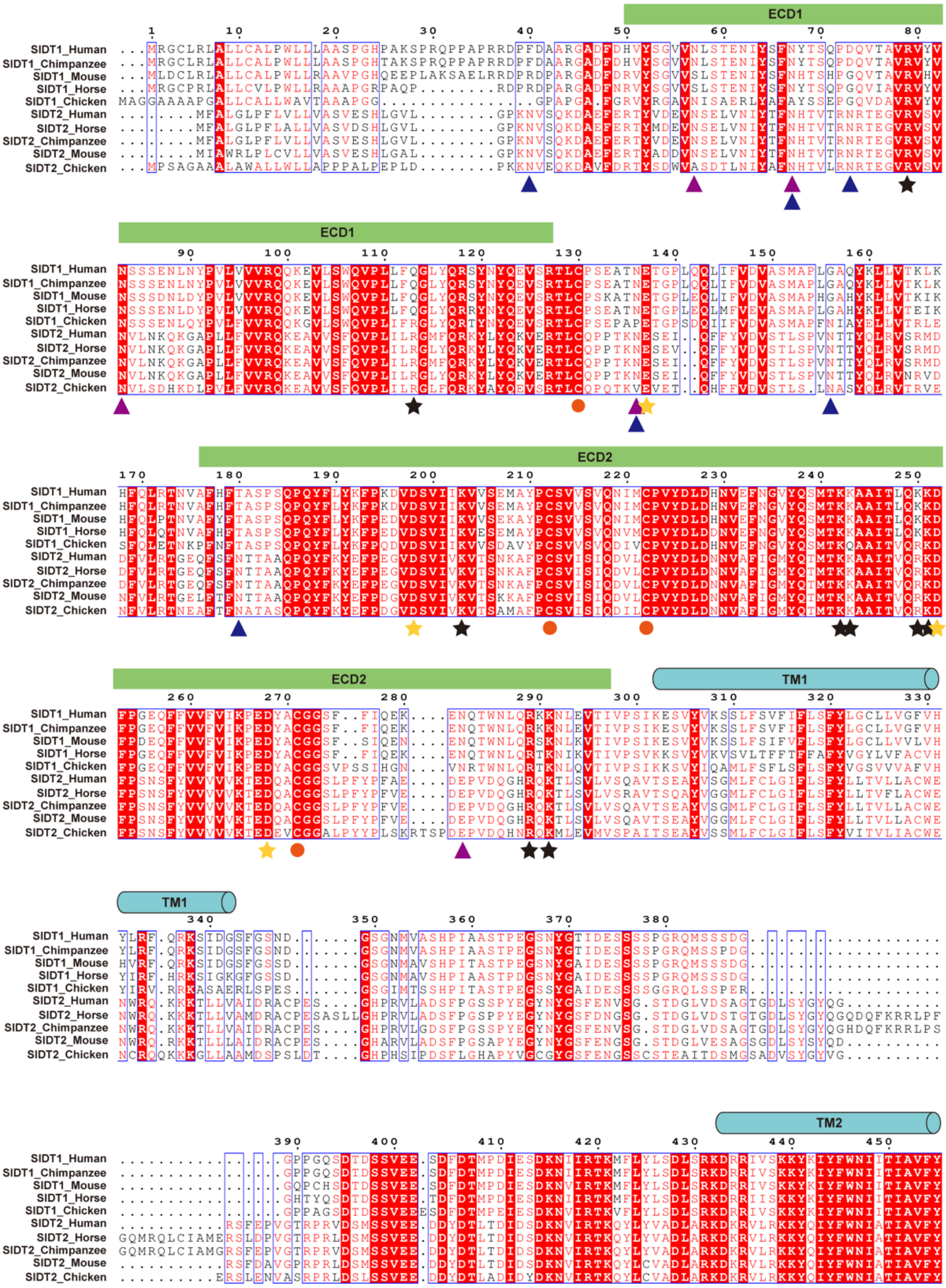

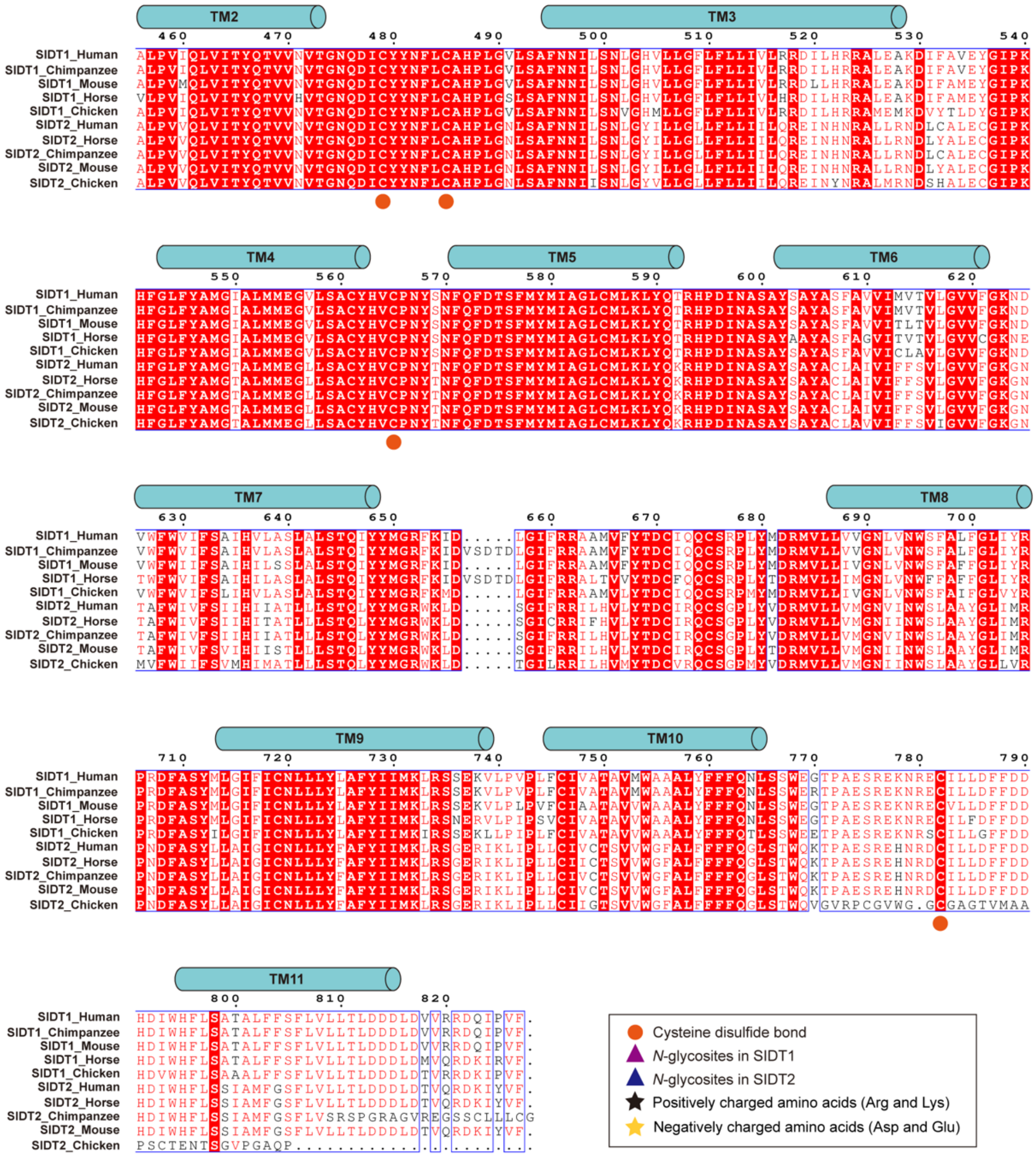

Supplementary information, Fig. S5 | Sequence alignment of SIDT1 and SIDT2 with their homologs. The primary sequences of different species of SIDT1 and SIDT2 are compared using ClustalW[15] and visualized using ESPript3.0[16]. The secondary structural elements of human SIDT1 predicted by AlphaFold[17] are indicated above the sequence alignment. Identically conserved residues are shaded red. Symbols below the alignment indicate cysteines for disulfide bond formation (orange circles), *N*-glycosites in SIDT1 (violet triangles), *N*-glycosites in SIDT2 (deep blue triangles), positively charged amino acids (blank rectangles), and negatively charged amino acids (yellow rectangles). The UniProt IDs for the aligned sequences are: SIDT1_chimpanzee: H2QN47; SIDT1_mouse: Q6AXF6; SIDT1_horse: F6SIJ9; SIDT1_chicken: F1NBM4;

SIDT2_chimpanzee: H2Q4U5; SIDT2_mouse: Q8CIF6; SIDT2_horse: F6Y6L2; SIDT2_chicken: A0A8V0ZU80.

Supplementary information, Fig. S6

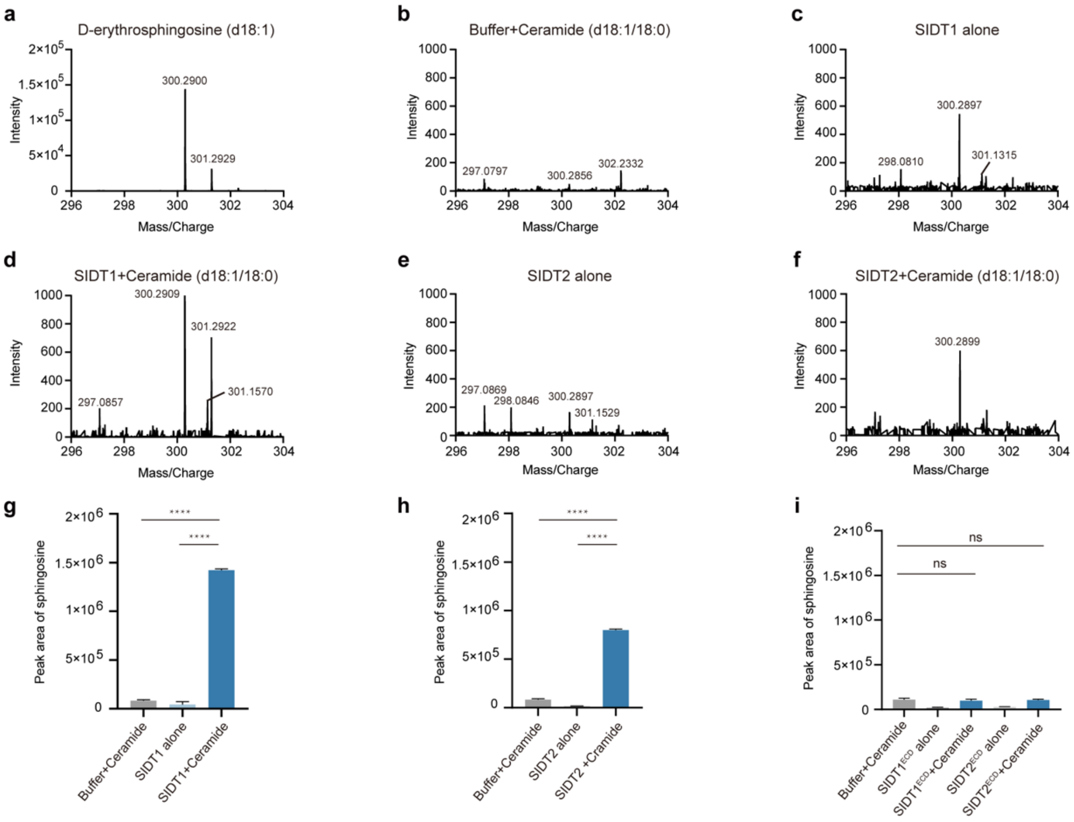

Supplementary information, Fig. S6 | LC-MS/MS analysis of the ceramidase activity of SIDT1 and SIDT2. **a-f** Representative mass spectrum for sphingosine detection is shown. The d-erythrosphingosine (d18:1) was used as the standard (**a**). Representative mass spectrum for the blank buffer plus ceramide (d18:1/18:0) (**b**), SIDT1 alone (**c**), SIDT1 plus ceramide (d18:1/18:0) (**d**), SIDT2 alone (**e**), and SIDT2 plus ceramide (d18:1/18:0) (**f**) are shown, respectively. The ceramidase activity of SIDT1 and SIDT2 was quantified by comparing the mass weight with a sphingosine standard. **g-i** Quantification of the ceramidase activity of SIDT1 (**g**), SIDT2 (**h**) and SIDT1/2^{ECD} (**i**) was performed using d-erythrosphingosine (d18:1) as a standard through high-resolution LC-MS/MS analysis.

Supplementary information, Fig. S7

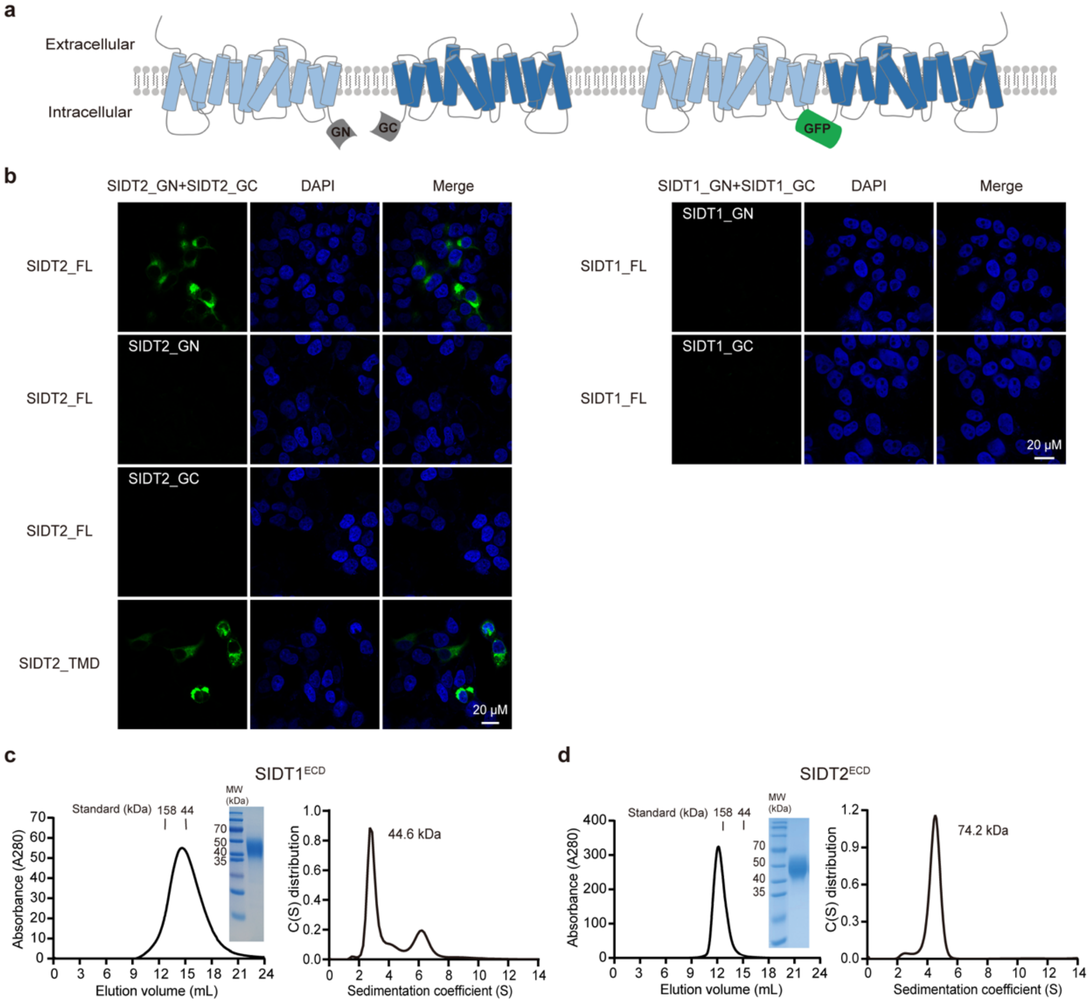

Supplementary information, Fig. S7 | SIDT1 and SIDT2 exhibit oligomeric states *in situ* and *in vitro*. a Schematic diagram representing the principle of the BiFC assay. **b** High-resolution confocal images of HEK293T cells expressing SIDT2 (FL: full-length, top; TMD, bottom) fused to the N-terminal and C-terminal fragments of GFP fluorescent protein. The SIDT1/2_GN and SIDT1/2_GC were used as negative controls. Scale bar, 20 µm. **c, d** Characterization of the purified SIDT1^{ECD} (**c**) and SIDT2^{ECD} (**d**). The left panel displays the SEC of purified SIDT1^{ECD} using a Superdex 200 increase 10/300 GL column and the SDS-PAGE analysis of peak fractions of interest. The right panel shows SV-AUC analysis of the molecular weight of SIDT1^{ECD} (**c**) and SIDT2^{ECD} (**d**) in solution. These SV-AUC experiments were performed twice with equivalent results. Values in the panel represent mean ± s.d.

Supplementary information, Fig. S8

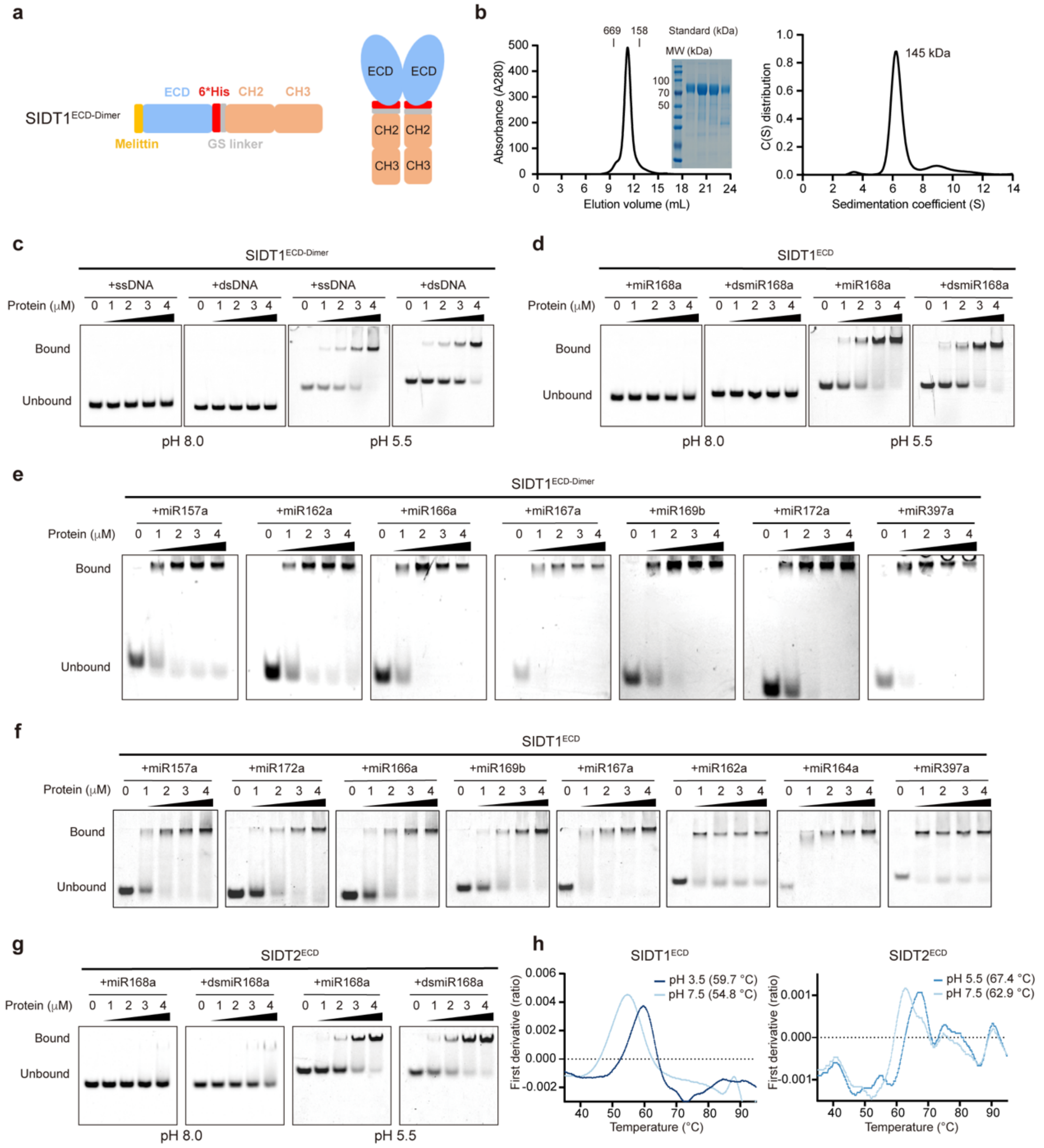

Supplementary information, Fig. S8 | The ECDs of SIDT1 and SIDT2 bind to small RNAs under acidic conditions. a Schematic diagram of a fusion protein of SIDT1^{ECD} with immunoglobulin fragment (Fc; SIDT1$^{ECD\text{-}Dimer}$). **b** Characterization of the purified SIDT1$^{ECD\text{-}Dimer}$. Left panel: Chromatogram of purified SIDT1 $^{ECD\text{-}Dimer}$ via a Superose 6 Increase 10/300 column. SDS-PAGE gel (Coomassie stained) displays proteins in peak fractions. The calibration standard for gel filtration chromatography is a mixture of thyroglobulin, γ-globulin, and ovalbumin proteins with approximate molecular weights of 669 kDa (13.0 mL), 158 kDa (16.4 mL), and 44 kDa (17.5 mL), respectively. Right panel: SV-AUC analysis of the molecular weight of SIDT1$^{ECD\text{-}Dimer}$

in solution. This SV-AUC assay was performed twice with equivalent results. Values in the panel represent mean ± s.d. **c-g** The DNA binding activities of SIDT1$^{ECD\text{-}Dimer}$ (**c**) and the miRNAs binding activities of both SIDT1^{ECD} (**d**) and SIDT2^{ECD} (**g**) were examined under pH 5.5 and pH 8.0. An array of plant-derived miRNA-binding activities of SIDT1$^{ECD\text{-}Dimer}$ (**e**) and SIDT1^{ECD} (**f**) were revealed by EMSA at pH 5.5. The same EMSA setups as presented in **Fig. 1** were used in these experiments. Bound, protein-miRNA complexes; Unbound, free miRNAs. **h** Protein stability of SIDT1^{ECD} and SIDT2^{ECD} was monitored in real-time as the temperature increased from 35 to 95 °C. DSF profiles (ratio between fluorescence at 350 nm and 330 nm) are displayed. DSF experiments were performed three times, and a representative result is shown.

Supplementary information, Fig. S9

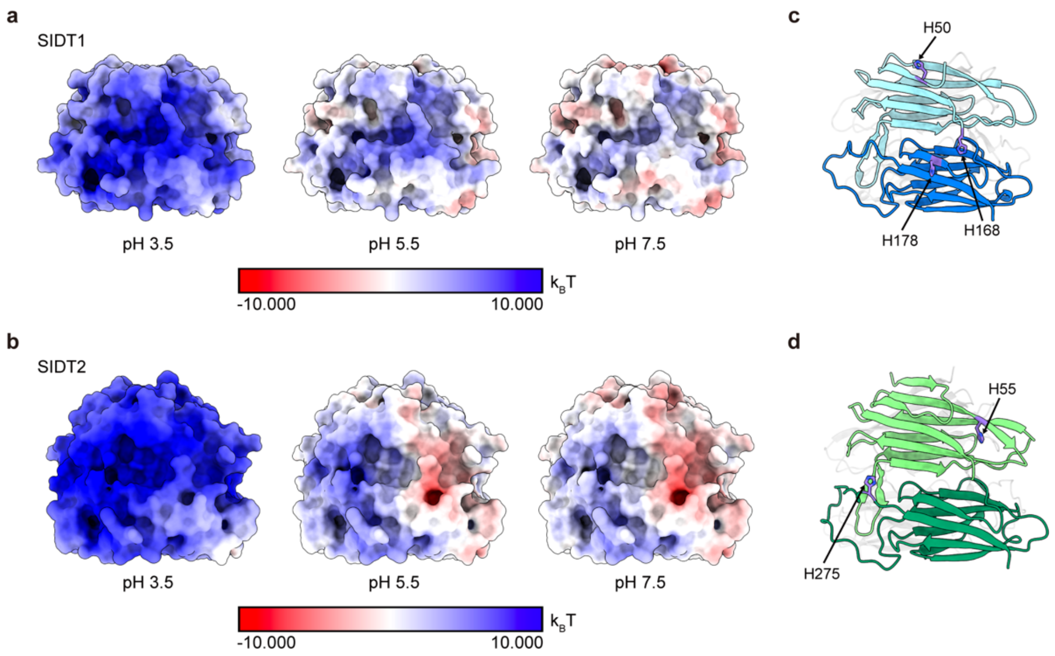

Supplementary information, Fig. S9 | The electrostatic potentials of SIDT1 and SIDT2. The electrostatic potentials of SIDT1 (**a**) and SIDT2 (**b**) at different pH values (pH 3.5, 5.5 and 7.5) are mapped onto their solvent-accessible surfaces. The electrostatic potentials are colored from red (negative charge) to blue (positive charge) in the range of −10.0 to 10.0 k_BT. The electrostatic potentials of SIDT1 and SIDT2 were calculated using the APBS-PDB2PQR software suite[18]. **c, d** The presence of histidine residues at the protein/RNA interface of SIDT1 (**c**) and SIDT2 (**d**) may contribute to the observed pH-dependent affinity. Histidine residues are shown as purple sticks.

Supplementary information, Fig. S10

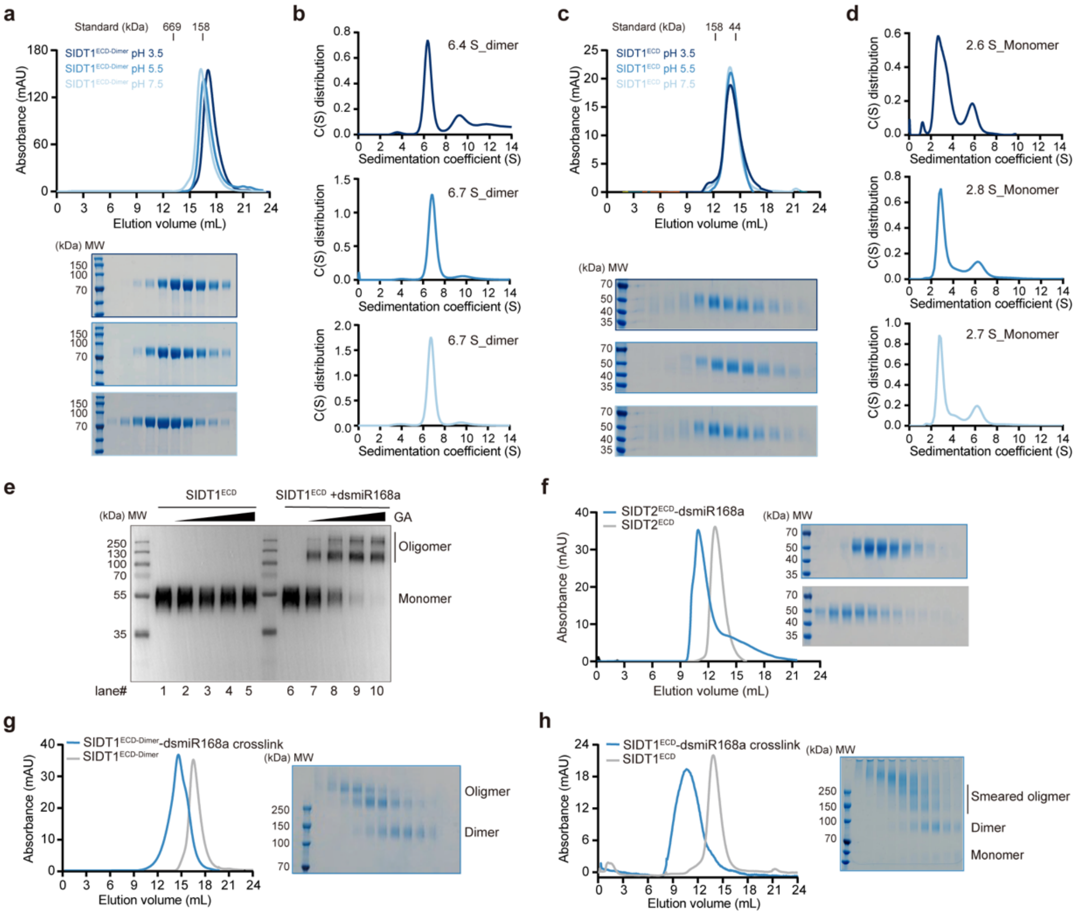

Supplementary information, Fig. S10 | Small RNAs promote ECD oligomerization under acidic conditions. a-d SEC and SV-AUC analysis of the oligomeric state of SIDT1$^{\text{ECD-Dimer}}$ and SIDT1^{ECD} at pH 3.5 (deep blue), pH 5.5 (blue), and pH 7.5 (light blue), respectively. SV-AUC analysis of the sedimentation coefficient corresponding to SEC results. SV-AUC experiments were performed twice with equivalent results. Values in the panel represent mean ± s.d. **e** Characterization of the oligomeric state of SIDT1^{ECD} via cross-linking assay. The cross-linking using glutaraldehyde (GA) and final products were analyzed by Western blot. Lanes 1 to 5 (without dsmiR168a) and 6-10 (with dsmiR168a) correspond to 30 minutes of incubation GA at pH 5.5 with at the following concentrations: 0%, 0.05%, 0.1%, 0.2%, and 0.5%. **f** The dsmiR168a triggers the assembly of SIDT2^{ECD} into oligomer, as determined by SEC. **g, h** SEC profiles of cross-linked SIDT1$^{\text{ECD-Dimer}}$ and SIDT1^{ECD} with dsmiR168a, non-reducing

SDS-PAGE (**g**) or standard SDS-PAGE (**h**) analysis of peak fractions of interest, respectively.

Supplementary information, Fig. S11

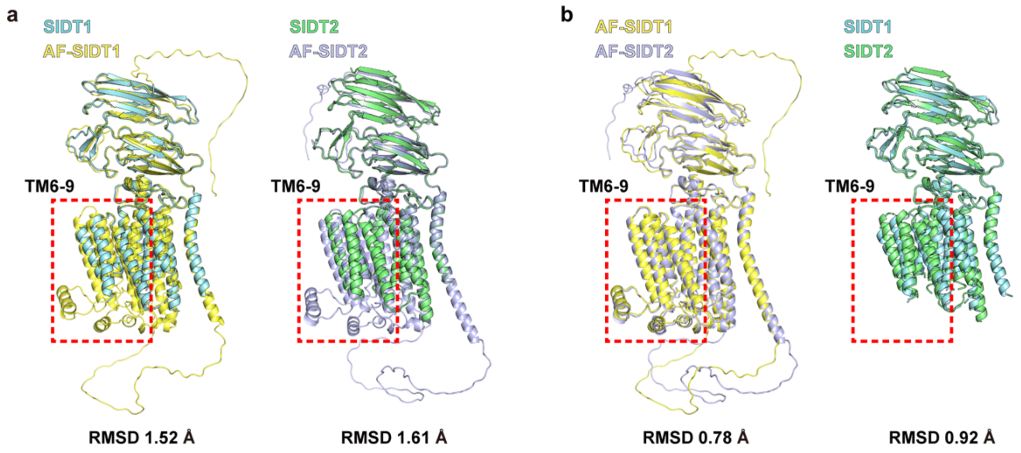

Supplementary information, Fig. S11 | Structural comparison of the cryo-EM determined structures with the AlphaFold predicted structures. a Comparison of experimental SIDT1 and SIDT2 models with their AlphaFold predicted AF-SIDT1 (left) and AF-SIDT2 (right) models, respectively. **b** Comparison between AlphaFold predicted models (left) and experimental models (right), respectively. RMSD, root-mean-square deviation.

Supplementary information, Fig. S12

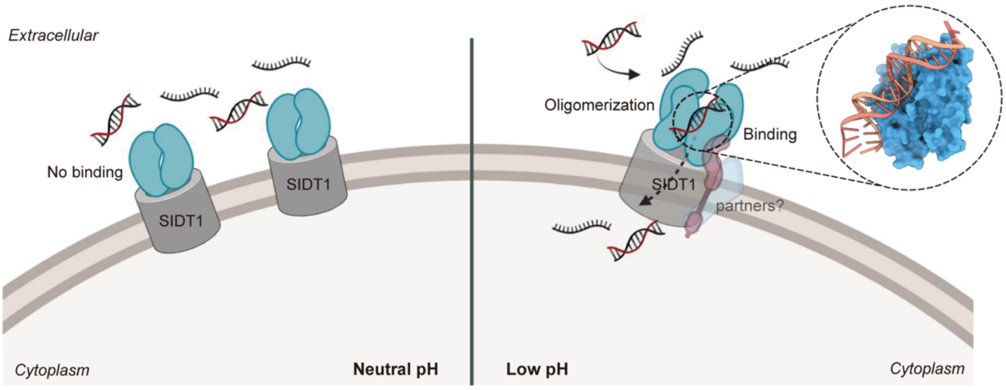

Supplementary information, Fig. S12 | Possible model depicting pH-dependent recognition by SIDT1 and their potential involvement in small RNAs transport. This model is also applicable to SIDT2.

Supplementary information, Table S1 | Cryo-EM data collection, refinement and validation statistics.

	SIDT1 dimer			SIDT2 dimer		
**Data collection and processing**						
Magnification	29,000×	105,000×		29,000×	165,000×	
Voltage (kV)	300	300		300	300	
Electron exposure (e⁻/Å^2)	60	60		60	60	
Defocus range (μm)	-1.2 to -2.0	-1.2 to -1.8		-1.2 to -2.0	-1.2 to -1.8	
Pixel size (Å)	0.82	1.35		0.82	0.82	
Symmetry imposed	C2	C2		C2	C2	
Initial particle projections (no.)	3,051,684	802,519		1,694,054	1,470,320	
Sub-maps	Map 1 SIDT1 overall	Map 2 SIDT1 ECD local-refinement	Map 3 SIDT1 TMD local-refinement	Map 1 SIDT2 overall	Map 2 SIDT2 ECD local-refinement	Map 3 SIDT2 TMD local-refinement
Final particle images (no.)	149,089	94,868	81,463	201,729	187,510	67,365
Map resolution (Å)	3.33	3.47	3.53	3.17	3.27	3.33
FSC threshold	0.143	0.143	0.143	0.143	0.143	0.143
**Refinement**						
Model composition						
Non-hydrogen atoms	9078	4176	4902	9478	4402	5592
Protein residues	1118	508	610	1176	526	714
Ligands	6	6	0	16	16	0
B factors (Å^2, mean value)	61.78	51.08	154.30	45.73	45.28	73.80
Protein	84.51	87.73	N/A	71.71	68.67	N/A
Ligand						
R.m.s. deviations						
Bond lengths (Å)	0.003	0.004	0.003	0.005	0.04	0.06
Bond angles (°)	0.570	0.684	0.724	0.994	0.951	0.960
Validation						
MolProbity score	1.55	1.69	1.58	1.61	1.57	1.54
Clash score	9.59	10.66	11.65	10.91	11.41	10.45
Poor rotamers (%)	0.40	0.85	0.57	0.39	0.42	0.50
Ramachandran plot						
Favored (%)	97.81	97.22	98.63	97.74	98.47	99.14
Allowed (%)	2.19	2.78	1.37	2.26	1.53	0.86
Disallowed (%)	0.00	0.00	0.00	0.00	0.00	0.00

Supplementary information, Table S2 | Sequences of plant-derived miRNAs prevalent in the serum of healthy Chinese individuals[19,20].

Name	sequence
miR157a	UUGACAGAAGAUAGAGAGCAC
miR162a	UCGAUAAACCUCUGCAUCCAG
miR164a	UGGAGAAGCAGGGCACGUGCA
miR166a	UCGGACCAGGCUUCAUUCCCC
miR167a	UGAAGCUGCCAGCAUGAUCUA
miR168a	UCGCUUGGUGCAGAUCGGGAC
miR169b	CAGCCAAGGAUGACUUGCCGG
miR172a	AGAAUCUUGAUGAUGCUGCAU
miR397a	UCAUUGAGUGCAGCGUUGAUG

References

1. Huang, X. et al. *Progress in Biophysics and Molecular Biology* **156**, 3-13 (2020).
2. Mastronarde, D.N. *J Struct Biol* **152**, 36-51 (2005).
3. Zheng, S.Q. et al. *Nature Methods* **14**, 331-332 (2017).
4. Zhang, K. *Journal of Structural Biology* **193**, 1-12 (2016).
5. Scheres, S.H.W. *Journal of Structural Biology* **180**, 519-530 (2012).
6. Punjani, A. et al. *Nature Methods* **14**, 290-296 (2017).
7. Jumper, J. et al. *Nature* **596**, 583-589 (2021).
8. Pettersen, E.F. et al. *Journal of Computational Chemistry* **25**, 1605-1612 (2004).
9. Emsley, P. et al. *Acta Crystallographica Section D* **66**, 486-501 (2010).
10. Liebschner, D. et al. *Acta Crystallographica Section D* **75**, 861-877 (2019).
11. Kerppola, T.K. *Nature Protocols* **1**, 1278-1286 (2006).
12. Hellman, L.M. et al. *Nat Protoc* **2**, 1849-61 (2007).
13. Schuck, P. *Biophys J* **78**, 1606-19 (2000).
14. Bligh, E.G. et al. *Can J Biochem Physiol* **37**, 911-7 (1959).
15. Corpet, F. *Nucleic Acids Res* **16**, 10881-90 (1988).
16. Robert, X. et al. *Nucleic Acids Res* **42**, W320-4 (2014).
17. Jumper, J. et al. *Nature* **596**, 583-589 (2021).
18. Jurrus, E. et al. *Protein Science* **27**, 112-128 (2018).
19. Zhang, L. et al. *Cell Res* **22**, 107-26 (2012).
20. Kozomara, A. et al. *Nucleic Acids Res* **47**, D155-d162 (2019).
